# Frequency analysis of TRBV subfamily sjTRECs to characterize T-cell reconstitution in acute leukemia patients after allogeneic hematopoietic stem cell transplantation

**DOI:** 10.1186/1756-8722-4-19

**Published:** 2011-04-23

**Authors:** Xiuli Wu, Kanger Zhu, Xin Du, Shaohua Chen, Lijian Yang, Jufeng Wu, Qifa Liu, Yangqiu Li

**Affiliations:** 1 Institute of Hematology, Medical College, Jinan University, Guangzhou 510632, PR China; 2 Department of Hematology, Nanfang Hospital, Southern Medical University, Guangzhou 510515, PR China; 3 Department of Hematology, Guangdong General Hospital, Guangzhou 510080, PR China; 4 Department of Hematology, Hainan Province People's Hospital, Haikou 570311, PR China

## Abstract

**Background:**

Allogeneic hematopoietic stem cell transplantation (allo-HSCT) leads to a prolonged state of immunodeficiency and requires reconstitution of normal T-cell immunity. Signal joint T-cell receptor excision DNA circles (sjTRECs) are markers of developmental proximity to the thymus that have been used to evaluate thymic function related to T-cell immune reconstitution after HSCT. To assess the proliferative history in different T-cell receptor beta variable region (TRBV) subfamilies of T cells after HSCT, expansion of TRBV subfamily-naive T cells was determined by analysis of a series of TRBV-BD1 sjTRECs.

**Methods:**

sjTRECs levels were detected by real-time quantitative polymerase chain reaction (PCR) in peripheral blood mononuclear cells (PBMCs) from 43 Chinese acute leukemia patients who underwent allo-HSCT. Twenty-three TRBV-BD1 sjTRECs were amplified by semi-nested PCR. Sixteen age-matched healthy volunteers served as normal controls.

**Results:**

sjTRECs levels were low or undetectable in the first 6 weeks after allo-HSCT and increased after 8 weeks post HSCT; however, sjTRECs levels at week 20 post-HSCT were still less than normal controls. Frequencies of TRBV subfamily sjTRECs in PBMCs from recipients at week 8 post-HSCT (29.17 ± 20.97%) or at week 16 post-HSCT (38.33 ± 9.03%) were significantly lower than those in donors (47.92 ± 13.82%) or recipients at pre-HSCT (45.83 ± 14.03%). However, frequencies of TRBV subfamily sjTRECs in recipients at week 30 post-HSCT (42.71 ± 21.62%) were similar to those in donors and recipients at pre-HSCT. sjTRECs levels in donors had a positive linear correlation with sjTRECs levels in recipients within 8-12 weeks post-HSCT. Patients with acute graft-versus-host disease (GVHD) or chronic GVHD had profoundly reduced TRECs levels during the first year post-HSCT. Frequencies of BV22-BD1 sjTRECs and BV23-BD1 sjTRECs in patients with GVHD were significantly lower than those in recipients at pre-HSCT, and the frequencies of BV22-BD1 sjTRECs in patients with GVHD were significantly lower than those in donors.

**Conclusions:**

Reconstitution of thymic output function resulted in a period of immunodeficiency, with low or undetectable TRECs after transplantation, although fludarabine-based dose-reduced conditioning regimens were used. GVHD could affect reconstitution of thymic output function and reduce sjTRECs levels and frequencies of TRBV-BD1 sjTRECs. Low frequency of BV22-BD1 and BV23-BD1 sjTRECs might be associated with GVHD.

## Background

Allogeneic hematopoietic stem cell transplantation (allo-HSCT) provides a potentially curing treatment for refractory hematopoietic malignancies and is often the only available treatment for acute leukemia. The transplantation procedure/conditioning regimen generally leads to a prolonged state of immunodeficiency, characterized by persistent low levels of naïve T cells. Successful allo-HSCT requires reconstitution of normal T-cell immunity. The T-cell population can be regenerated through two different pathways [1]. The thymic-independent pathway involves expansion of graft-derived mature donor T cells, whereas the thymic-dependent pathway involves regeneration of T cells with a more diverse T-cell receptor (TCR) repertoire from graft-derived precursor cells. Because thymic function is necessary for de novo generation of T cells after transplantation, quantification of T-cell receptor excision DNA circles (TRECs) in peripheral blood T cells can be used to determine the potential function of T lymphopoiesis after HSCT [2]. Signal joint T-cell receptor excision DNA circles (sjTRECs) are the products of rearrangement of the T-cell receptor gene, leading to the excision of circular DNA fragments from genomic DNA during thymocyte development. Quantification of sjTRECs in peripheral blood, as a measure of thymic function, overcomes the disadvantages associated with the use of T-cell surface molecules, such as CD45RA, as markers for recent thymic emigrants (RTEs). Thus, sjTRECs are markers of developmental proximity to the thymus and their concentrations in peripheral blood can be used to estimate thymic output and evaluate thymic function in patients after stem cell transplantation.

Graft-versus-host disease (GVHD) is a major complication following allo-HSCT [3-5]. Poor reconstitution of T-cell immunity (including reconstitution of recent thymic output function) has been associated with GVHD. GVHD may predict low TRECs levels and slow naïve T-cell recovery [6,7]. However, most previously published studies have focused only on the total number of RTEs, as measured by quantitative analysis of total sjTRECs. This approach does not consider the complexity of thymic output and T-cell proliferation in different TRBV subfamilies, which is an important factor in immune competence. To assess the proliferative history in different TRBV subfamilies of T cells, expansion of particular TRBV subfamily T cells has been recently determined by quantitative analysis of a series of TRBV-BD1 sjTRECs [8-11]. However, T-cell proliferation in different TRBV subfamilies after allo-HSCT remains poorly understood.

The main objective of the present study was to investigate reconstitution of recent thymic output function after allo-HSCT through analysis of total sjTRECs and TRBV subfamily sjTRECs. Analysis of TRBV subfamily sjTRECs frequencies may be beneficial for evaluating T-cell reconstitution in acute leukemia patients after allo-HSCT and may further support and explain reconstitution of RTEs measured by quantitative detection of total sjTRECs.

## Materials and methods

### Patients

Forty-three acute leukemia patients (median age, 30.6 ± 10.2 years; range, 17-52 years; classified according to the French-American-British (FAB) criteria as 27 cases of acute lymphocytic leukemia (ALL) and 16 cases of acute myeloid leukemia (AML)) underwent allo-HSCT. All patients had received fludarabine-based, dose-reduced conditioning regimens (including low-dose fludarabine 30 mg/m^2^·d × 3-5 d; total dose 90-150 mg/m^2^) and were full donor chimeras in remission. Transplanted cells were obtained from the bone marrow or peripheral blood of an HLA genotypically identical sibling (median age, 32.1 ± 8.2 years; range, 20-49 years). No specific procedure was performed to enrich or deplete a specific cell population. Acute GVHD (aGVHD) and chronic GVHD (cGVHD) were diagnosed and graded as described previously [12]. Peripheral blood was obtained from 16 age-matched healthy volunteers (median age, 30.8 ± 7.6 years; range, 17-48 years). Patient blood samples were collected at pre-HSCT and every 2 weeks after allo-HSCT and at GVHD onset, and subsequently peripheral blood mononuclear cells (PBMCs) were separated from freshly drawn anticoagulated blood using Ficoll-Hypaque density gradient centrifugation. All procedures were conducted in accordance with the guidelines of the Medical Ethics Committees of the health bureau of Guangdong Province, China. Samples were collected with informed consent.

### Flow cytometry

The following fluorescein isothiocyanate (FITC) - or phycoerythrin (PE) - labeled monoclonal antibodies were used: mouse anti-human CD4, CD8, CD45RA, and CD45RO (BD BioSciences, USA). Stainings were performed by incubating cells with the appropriate pool of antibodies for 30 min at 4°C followed by a series of washes with phosphate-buffered saline solution supplemented with 2% fetal calf serum. Isotype-matched FITC-labeled mouse IgG served as the negative control.

### DNA extraction

Total DNA from distinct cell populations was extracted using the QIAamp DNA Blood Mini Kit (Qiagen, Germany). The quality of DNA was analyzed in 1% agarose gels stained with ethidium bromide, and the concentration was determined by spectrophotometric analysis at 260 and 280 nm (Lambda 45 UV/VIS Spectrometer; Perkin Elmer, USA).

### Quantification of sjTRECs by real-time polymerase chain reaction (PCR)

The sjTRECs levels were detected by quantitative real-time PCR. DNA extraction of PBMCs was performed using the QIAprep Spin Miniprep Kit (Qiagen, Germany). To precisely determine the percentage of cells carrying sjTRECs, we used a duplex vector that included a fragment of the δRec-ψJα sjTREC and a fragment of the RAG2 gene, constructed by Prof. C.A. Schmidt [13,14]. RAG2 was first cloned in the T-A acceptor site, and subsequently the TREC was cloned into the *Eco*R V restriction site of the TOPO TA vector. Based on the DNA concentration, standard dilutions of the vector from 10^7 ^to 10^1 ^copies were prepared. Briefly, 50-μL PCR reactions were performed with approximately 100 ng of genomic DNA, 25 pmol of each primer, 10 nmol of each dNTP, 1.25 U of AmpliTaq Gold polymerase, 5 pmol of 6-FAM-TAMRA probe, and PCR buffer with 4.5 mM MgCl_2_. After an initial denaturation at 95°C for 5 min, 45 cycles consisting of 95°C for 30 s and 67°C for 1 min were performed. The amplification was performed on MJ Research DNA Engine Opticon 2 PCR cycler (BIO-RAD, USA).

### Semi-nested PCR

Twenty-three TRBV-BD1 sjTRECs were amplified by semi-nested PCR using 0.325 μg of genomic DNA, corresponding to 5 × 10^4 ^PBMCs. Two nested 5'-TRBD1 primers, located upstream of the segment, and 23 BV primers (BV1-19 and BV21-24; rearrangement of BV20 does not generate a sjTREC because of its reverse orientation) were used [14]. In the first-round PCR, aliquots of the DNA (2 μl) were amplified in 10-μl reactions with one of the 23 BV primers (antisense) and a BD1 primer (sense primer); the final reaction mixture contained 0.375 μM of sense and antisense primers, 0.1 mM of dNTPs, 1.5 mM MgCl_2_, 1 × PCR buffer, and 1 U of Taq polymerase (Promega, USA). Amplification was performed as described previously [14].

### Statistical analyses

The correlation of sjTRECs levels between pre-HSCT and post-HSCT and that of sjTRECs levels between donors and recipients after allo-HSCT were analyzed using the Pearson correlation test. The Mann-Whitney *U*-test was used to compare the difference in levels or frequencies of sjTRECs or TRBV-BD1 sjTRECs. The Fisher exact test was used to compare the frequency of TRBV-BD1 sjTRECs in PBMCs between patients at GVHD onset and patients at pre-HSCT or donors. Data were analyzed using the SPSS software (ver. 13.0) and differences were considered statistically significant when the *P*-value was less than 0.05.

## Results

### RTEs of healthy controls, donors, and recipients

In the present study, donors and normal controls were of similar age to the recipients, with ages ranging mostly from 20 to 35 years. We found no significant correlation between sjTRECs levels and age in the healthy controls, donor group, or recipient group at pre-HSCT (*r *= -0.001, -0.110, -0.232, respectively; *P *= 0.998, 0.664, 0.286, respectively) and no significant age-associated correlation of the numbers of the TRBV-BD1 sjTRECs subfamily in the healthy controls, donor group, or recipient group (*r *= -0.591, 0.455, 0.543, respectively; *P *= 0.072, 0.441, 0.457, respectively). No significant correlation was found between the sjTRECs levels after allo-HSCT and age of the recipients (*r *= -0.197; *P *= 0.107), or between the numbers of the TRBV-BD1 sjTRECs subfamily after allo-HSCT and age of recipients (*r *= 0.422; *P *= 0.071).

The sjTRECs levels in PBMCs from healthy controls (3.011 ± 0.838 copies per 1000 PBMCs) were higher than those in the donor group (1.299 ± 1.573 copies per 1000 PBMCs) and in the recipients group at pre-HSCT (1.367 ± 2.102 copies per 1000 PBMCs) (*P *= 0.000, 0.000, respectively). No statistical correlation was found between sjTRECs levels in recipients at pre-HSCT and those within 12 weeks post-HSCT (including the ~4 weeks post-HSCT group, 4-8 weeks post-HSCT group, and 8-12 weeks post-HSCT group) (*r *= -0.197, 0.527, -0.214, respectively; *P *= 0.562, 0.145, 0.527, respectively). No statistical correlation was also found between sjTRECs levels in donors and the sjTRECs levels within 8 weeks post-HSCT (including the ~4 weeks post-HSCT group and the 4-8 weeks post-HSCT group) (*r *= -0.153, -0.160; *P *= 0.771, 0.638). However, the sjTRECs levels in donors showed a positive linear correlation with the sjTRECs levels in recipients within 8-12 weeks post-HSCT (*r *= 0.869; *P *= 0.011).

### Reconstitution of recent thymic output function in the early period after allo-HSCT

**Figure 1:**
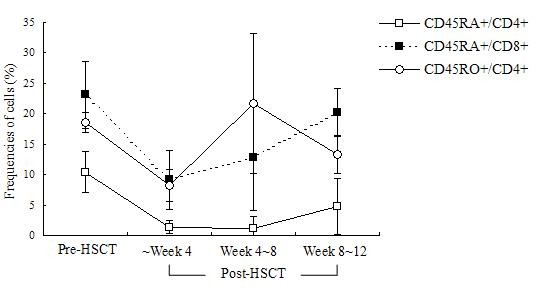
**Frequencies of T lymphocyte subsets**. Error bars represent the standard error of the mean (SEM).

The changes in frequencies of CD45RA^+^/CD4^+^, CD45RA^+^/CD8^+^, and CD45RO^+^/CD4^+ ^T cells after HSCT are shown in Figure 1. In the early period after HSCT (within 12 weeks), the frequencies of CD45RA^+^/CD4^+^, CD45RA^+^/CD8^+^, and CD45RO^+^/CD4^+ ^T cells in patients at week 4 post-HSCT were significant lower than those at pre-HSCT (*P *= 0.000). The frequencies of CD45RA^+^/CD4^+ ^T cells remained at low levels within 8 weeks after HSCT, and higher after week 12 post-HSCT (*P *= 0.003). Within 8 weeks post-HSCT, the CD45RO^+ ^T cells that expanded were predominant, but after week 8 post-HSCT, CD45RA^+^/CD8^+ ^T cells predominated over CD45RO^+ ^T cells in PBMCs (*P *= 0.000).

**Figure 2:**
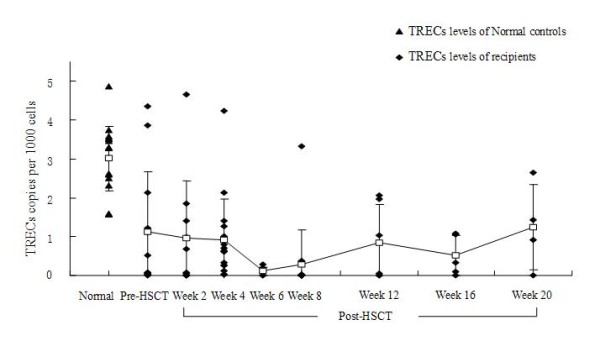
**Changes of sjTRECs levels in the early period after allo-HSCT**. The sjTRECs levels of recipients at pre-HSCT were lower than the sjTRECs levels of the normal controls. The sjTRECs levels were near the baseline at week 6 post-HSCT and increased after 8 weeks post-HSCT. But the sjTRECs levels at week 20 after HSCT were still lower than the normal controls. Error bars represent the SEM. The squares represent the mean levels and the folding line represents the trend.

The sjTRECs levels were low or undetectable in the first 6 weeks after allo-HSCT (Figure 2). The mean sjTRECs levels were lowered from 0.971 ± 1.462 copies per 1000 PBMCs at week 2 to 0.918 ± 1.055 copies per 1000 PBMCs at week 4, and near baseline at week 6 (0.107 ± 0.108 copies per 1000 PBMCs) after transplantation. The sjTRECs levels increased after week 8 post-HSCT. The sjTRECs levels at week 20 after allo-HSCT (1.247 ± 1.100 copies per 1000 PBMCs) were similar to the sjTRECs levels at pre-HSCT (1.119 ± 1.549 copies per 1000 PBMCs; *P *= 0.870); however, they were still lower than the normal controls (3.011 ± 0.838 copies per 1000 PBMCs; *P *= 0.001). Additionally, four recipients (three cases of AML and one case of ALL) had an early relapse after allo-HSCT, and their sjTRECs levels in PBMCs returned to the baseline or were undetectable (their sjTRECs levels before allo-HSCT were 1.028, 4.035, 3.122, and 0.027 copies per 1000 PBMCs, respectively).

**Figure 3:**
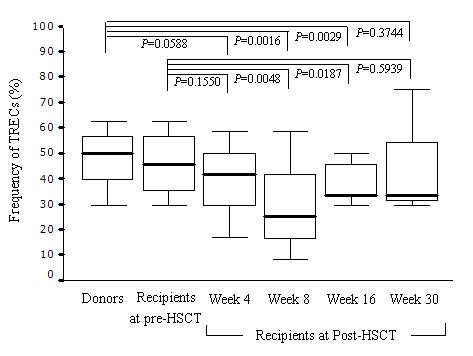
**Frequencies of TRBV-BD1 sjTRECs at the 5 × 10^4 ^PBMC level after allo-HSCT**. Error bars represent the SEM.

Samples were amplified to estimate the frequency of TRBV-BD1 sjTRECs and sequences of the junction regions of each TRBV-BD1 sjTRECs were confirmed by direct sequencing of PCR products (data not shown). Comparison of the frequencies of TRBV subfamily sjTRECs at the 5 × 10^4 ^PBMC level among donors, recipients at pre-HSCT, and recipients within 30 weeks post-HSCT (including the week 4 post-HSCT, week 8 post-HSCT, week 16 post-HSCT, and week 30 post-HSCT groups) revealed that the frequencies of TRBV subfamily sjTRECs in recipients at week 8 post-HSCT (29.17 ± 20.97%) or at week 16 post-HSCT (38.33 ± 9.03%) were significantly lower than in donors (47.92 ± 13.82%) or recipients at pre-HSCT (45.83 ± 14.03%; *P *< 0.05). The frequency of TRBV subfamily sjTRECs in recipients at week 30 post-HSCT (42.71 ± 21.62%) was similar to that in donors or recipients at pre-HSCT (Figure 3). Low frequencies of particular TRBV subfamily sjTRECs were found in recipients at pre-HSCT (BV2-BD1, BV3-BD1, BV7-BD1, BV8-BD1, BV9-BD1, BV12-BD1, and BV17-BD1 sjTRECs), in the week 4 post-HSCT group (BV7-BD1, BV9-BD1, BV12-BD1, BV17-BD1, and BV18-BD1 sjTRECs), in the week 8 post-HSCT group (BV2-BD1, BV3-BD1, BV7-BD1, BV9-BD1, BV12-BD1, BV17-BD1, BV22-BD1, and BV23-BD1 sjTRECs), in the week 16 post-HSCT group (BV1-BD1, BV3-BD1, BV5-BD1, BV7-BD1, BV9-BD1, BV12-BD1, and BV22-BD1 sjTRECs), and in the week 30 post-HSCT group (BV23-BD1 sjTRECs).

### Changes in the recent thymic output function with GVHD

The sjTRECs levels were measured in patients who had no episodes of GVHD and patients at acute or chronic GVHD onset. As shown in Tables 1 and 2, the difference in sjTRECs levels between recipients with GVHD and recipients without GVHD within 2 years post-HSCT was statistically significant. The sjTRECs levels in patients with aGVHD or cGVHD were low or undetectable during the first year post-HSCT. With clinical immune treatment, sjTRECs levels in some cGVHD patients had increased after 2 years post-HSCT. Additionally, we found that one patient with immune treatment for cGVHD experienced a rise in sjTRECs levels (1.325 copies/1000 PBMCs) after 4 years post-HSCT.

**Table 1:** Relationship between aGVHD and sjTRECs levels after allo-HSCT

Groups	aGVHD	sjTRECs copies per 1000 PBMCs	P*
4 weeks post-HSCT	Yes	0.000 ± 0.000	0.000
	No	0.702 ± 1.153	
4-8 weeks post-HSCT	Yes	0.012 ± 0.037	0.003
	No	0.464 ± 0.626	
8-12 weeks post-HSCT	Yes	0.071 ± 0.139	0.036
	No	0.820 ± 1.121	

* Mann-Whitney *U*-test.

**Table 2:** Relationship between cGVHD and sjTRECs levels after allo-HSCT

Groups	cGVHD	sjTRECs copies per 1000 PBMCs	P*
4-6 months post-HSCT	Yes	0.032 ± 0.079	0.001
	No	1.487 ± 1.429	
6-12 months post-HSCT	Yes	0.248 ± 0.358	0.047
	No	1.426 ± 1.642	
2 years post-HSCT	Yes	0.573 ± 0.546	0.227
	No	0.835 ± 0.541	

* Mann-Whitney *U*-test.

**Figure 4:**
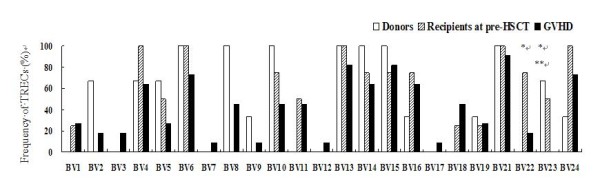
**Frequencies of 23 TRBV-BD1 sjTRECs subfamilies in PBMCs from patients with GVHD, donors, and recipients at pre-HSCT**. * *P *< 0.05, comparing patients with GVHD to recipients at pre HSCT. ** *P *< 0.05, comparing patients with GVHD to donors.

Comparison of the frequencies of 23 TRBV-BD1 sjTRECs among patients with GVHD, donors, and recipients at pre-HSCT showed that the frequencies of BV22-BD1 sjTRECs and BV23-BD1 sjTRECs in patients with GVHD were significantly lower than those in recipients at pre-HSCT (*P *= 0.039, 0.012), and the frequencies of BV22-BD1 sjTRECs in patients with GVHD were significantly lower than those in donors (*P *= 0.003). However, no significant difference was found in the frequencies of other TRBV-BD1 sjTRECs among groups of patients with GVHD and donors and recipients at pre-HSCT (*P *> 0.05; Figure 4).

## Discussion

During TCR rearrangement processes in the thymus, by-products in the form of sjTRECs are considered to be a valuable tool to estimate thymic function [14]. Quantitative analysis of δRec-ψJα sjTRECs provides information about total thymic output and TRBV-BD sjTRECs specific for each TRBV subfamily allow determination of the proliferative history of a particular TRBV subfamily [8-11]. In the present study, we detected both δRec-ψJα sjTRECs and TRBV-BD sjTRECs to evaluate not only the recent total naïve T-cell output but also the specific TRBV subfamily naïve T-cell output from the thymus in patients after HSCT.

The sjTRECs levels in recipients before allo-HSCT were lower than those in healthy controls, suggesting that recipients still had a low thymus output function before allo-HSCT. Also, sjTRECs levels in donors were lower than those in healthy controls. The cause may be that the blood samples of donors were collected after granulocyte colony-stimulating factor (G-CSF) mobilization, and G-CSF can influence T-cell immunity. Previous studies have indicated that age was a crucial factor determining the contribution of thymic output to T-cell recovery post-HSCT[6,7,15-18]. Patient age might be the single most important factor determining the success of immune reconstitution post-HSCT and whether thymic-dependent or -independent pathways contribute to T-cell reconstitution post-HSCT. Thymic function and sjTRECs levels normally decrease with age. However, in the present study, we did not observe such a correlation between sjTRECs levels and age or between the numbers of TRBV-BD1 sjTRECs and age in healthy controls, the donor group, or the recipient group. Additionally, no statistical correlation was noted between the sjTRECs levels after allo-HSCT and age of recipients. The cause may be that the chosen ages of normal individuals, donors, and recipients mainly ranged from 20 to 35 years old, and for that narrow range of age, the immunological index of thymic function, such as sjTRECs levels or the numbers of TRBV-BD1 sjTRECs, demonstrates no significant age-associated correlation.

The early post-transplant period is characterized by profound immunodeficiency and recovery of a self-restricted, diverse T-cell repertoire is dependent on thymic production of T cells from hematopoietic progenitors. The appearance of sjTRECs after transplantation was associated with the emergence of phenotypically naïve T cells. Bahceci et al. measured the highest TRECs counts 2 weeks after non-myeloablative HSCT and observed a gradual decrease in TRECs numbers up to 6 months after HSCT, indicating that T-cell reconstitution was due rather to post-thymic T-cell expansion than to thymopoiesis [19]. However, Przybylski et al. [20] observed an increase in TRECs counts after an initial drop to undetectable levels, starting 2-3 months after HSCT and reaching a plateau 6 months after HSCT, indicating ongoing thymic output. Although fludarabine-based, non-myeloablative conditioning was performed in both studies, the regimen used in the Bahceci study (125 mg/m^2 ^fludarabine) was milder than that in the Przybylski study (180 mg/m^2 ^fludarabine). The differences in TRECs counts after HSCT might be due to different pre-transplantation conditioning. In the present study, we found that most recipients experienced a period of immunodeficiency with low or almost undetectable TRECs numbers at the early stage after transplantation, although all patients had received dose-reduced conditioning regimens (including low-dose fludarabine (90-150 mg/m^2^)). The sjTRECs levels were lowered, from 0.971 ± 1.462 copies per 1000 PBMCs at week 2 to 0.918 ± 1.055 copies per 1000 PBMCs at week 4, near baseline at week 6 after transplant, and increased after week 8. The sjTRECs levels at week 20 after allo-HSCT were elevated and similar to sjTRECs levels at pre-HSCT, but were still lower than the normal controls. We also found that CD45RA^+ ^T cells predominated over CD45RO^+ ^T cells in PBMCs after week 8 post-HSCT. These results confirmed that sjTRECs levels in PBMCs were restored in the short-term post-HSCT (within 12 weeks) via peripheral expansion of graft-derived mature T cells, and subsequently thymic-dependent T-cell recovery from graft-derived precursor cells predominated. Additionally, we found that sjTRECs levels in donors demonstrated a positive linear correlation with sjTRECs levels in recipients within 8-12 weeks post-HSCT. This corresponds to CD45RO^+ ^T-cell expansion, which predominated within 8 weeks post-HSCT. It also suggests that higher sjTRECs levels in donors should be beneficial to transplant recipients to rapidly reconstitute a functional immune system.

Most published studies of T-cell reconstitution have relied on post-transplantation measurement of TRECs and TRBV repertoire diversity [21]. Previous studies have focused only on the total number of RTEs, as measured by quantitative analysis of total sjTRECs. This approach does not examine the role of different TRBV subfamilies in T-cell proliferation and the complexity of thymic output. Additionally, analyzing the changes of the TRBV repertoire cannot indicate the source of the specific T-cell clones that came from the expansion of graft-derived mature donor T cells or the regeneration of T cells after thymic output from graft-derived precursor cells. To assess the proliferative history in different TRBV subfamilies of T cells, as in our previous study, we analyzed 23 subfamilies of TRBV-DB1 sjTRECs in AML patients and observed a significantly lower frequency of TRBV-DB1 sjTRECs [10]. In the present study, we observed that frequencies of TRBV subfamily sjTRECs in recipients at week 8 post-HSCT or at week 16 post-HSCT were significantly lower than those in donors or recipients at pre-HSCT. The frequencies of TRBV subfamily sjTRECs in recipients at week 30 post-HSCT were similar to those in donors or recipients at pre-HSCT, except that the TRBV23-BD1 subfamily sjTRECs remained at a low frequency. The results further support and explain the reconstitution of RTEs numbers in peripheral blood of acute leukemia patients after HSCT, as measured by quantitative detection of total sjTRECs.

GVHD has been demonstrated to have an adverse effect on thymic output, using sjTRECs to measure thymic output [22]. Przybylski et al. [20] found that recovery of TRECs after non-myeloablative allo-HSCT was not correlated with the onset of GVHD. Similarly, no effect of GVHD on TRECs was found in patients after non-myeloablative HSCT in Bahceci's research [19]. In the present study, sjTRECs levels were measured in patients who had no episode of GVHD or in patients at acute or chronic GVHD onset. The sjTRECs levels in patients with GVHD were low or undetectable for the first 6 months post-HSCT. Patients with acute GVHD or chronic GVHD had profoundly reduced sjTRECs levels during the first year post-HSCT. However, with clinical immune treatment, sjTRECs levels in some cGVHD patients could increase after 2 years post-HSCT. Notably, frequencies of BV22-BD1 and BV23-BD1 sjTRECs in patients with GVHD were significantly lower than those in recipients at pre-HSCT, and frequencies of BV22-BD1 sjTRECs in patients with GVHD were significantly lower than those in donors. These results indicated that GVHD could affect reconstitution of thymic output function and reduce sjTRECs levels and frequencies of TRBV-BD1 sjTRECs subfamilies, particularly BV22-BD1 and BV23-BD1 sjTRECs.

Previous studies had shown that the persistence of low sjTRECs numbers was associated with a higher incidence of GVHD [2,23], infection [6], and leukemic relapse [7]. Our study revealed that four recipients had early relapse after allo-HSCT and their sjTRECs levels in PBMCs returned to baseline or were undetectable, suggesting that sjTRECs could be a potentially relevant prognostic factor for acute leukemia patients who receive allo-HSCT.

In conclusion, analysis of the frequency of TRBV subfamily sjTRECs further support and coincide with quantitative detection of total sjTRECs, and whether low frequency of BV22-BD1 and BV23-BD1 sjTRECs subfamilies after HSCT might be associated with GVHD remains to be determined. Measuring and analyzing total sjTRECs levels and TRBV subfamily sjTRECs frequencies during immune reconstitution after HSCT would be useful to determine the status of thymic output function and ability of T-cell immune reconstitution more precisely, and may be beneficial in evaluating T-cell reconstitution in acute leukemia patients after allo-HSCT.

## Competing interests

The authors declare that they have no competing interests.

## Authors' contributions

WXL performed semi-nested PCR of TRBV-BD1 sjTRECs and data management; ZKE and DX and LQF provided the patients' samples. SHC, YLJ and WJF performed the RT-PCR and real-time PCR. YQL were responsible for the study design and data management. All authors read and approved the final manuscript.
